# Identification of high risk clinical and imaging features for intracranial artery dissection using high-resolution cardiovascular magnetic resonance

**DOI:** 10.1186/s12968-021-00766-9

**Published:** 2021-06-14

**Authors:** Zhang Shi, Xia Tian, Bing Tian, Zakaria Meddings, Xuefeng Zhang, Jing Li, David Saloner, Qi Liu, Zhongzhao Teng, Jianping Lu

**Affiliations:** 1 Department of Radiology, Changhai Hospital, Naval Medical University, 10 Building, 168 Changhai Road, Yangpu, Shanghai, 200433 China; 2 Department of Radiology, Addenbrooks’ Hospital, University of Cambridge, Cambridge, UK; 3 Department of Radiology and Biomedical Imaging, UCSF, San Francisco, CA USA; 4 Beijing Advanced Innovation Center for Biomedical Engineering, Beihang University, Beijing, China

**Keywords:** Intracranial artery dissection, Stroke, Cardiovascular magnetic resonance, MRI, Intramural hematoma

## Abstract

**Background:**

Intracranial artery dissection (IAD) often causes headache and cerebral vascular ischemic events. The imaging characteristics of IAD remain unclear. This study aims to characterize the appearance of culprit and non-culprit IAD using high-resolution cardiovascular magnetic resonance imaging (hrCMR) and quantify the incremental value of hrCMR in identifying higher risk lesions.

**Methods:**

Imaging data from patients who underwent intervention examination or treatment using digital subtraction angiography (DSA) and hrCMR using a 3 T CMR system within 30 days after the onset of neurological symptoms were collected. The CMR protocol included diffusion-weighted imaging (DWI), black blood T1-, T2- and contrast-enhanced T1-weighted sequences. Lesions were classified as culprit and non-culprit according to imaging findings and patient clinical presentations. Univariate and multivariate analyses were performed to assess the difference between culprit and non-culprit lesions and complementary value of hrCMR in identifying higher risk lesions.

**Results:**

In total, 75 patients were included in this study. According to the morphology, lesions could be classified into five types: Type I, classical dissection (n = 50); Type II, fusiform aneurysm (n = 1); Type III, long dissected aneurysm (n = 3); Type IV, dolichoectatic dissecting aneurysm (n = 9) and Type V, saccular aneurysm (n = 12). Regression analyses showed that age and hypertension were both associated with culprit lesions (age: OR, 0.83; 95% CI 0.75–0.92; p < 0.001 and hypertension: OR, 66.62; 95% CI 5.91–751.11; p = 0.001). Hematoma identified by hrCMR was significantly associated with culprit lesions (OR, 16.80; 95% CI 1.01–280.81; p = 0.037). Moreover, 17 cases (16 lesions were judged to be culprit) were diagnosed as IAD but not visible in DSA and 15 were Type I lesion.

**Conclusion:**

hrCMR is helpful in visualizing and characterizing IAD. It provides a significant complementary value over DSA for the diagnosis of IAD.

**Supplementary Information:**

The online version contains supplementary material available at 10.1186/s12968-021-00766-9.

## Background

Intracranial artery dissection (IAD) is the occurrence of a hematoma in an intracranial arterial wall induced by the split of its layers. It often causes headache and cerebral vascular ischemic events due to hemodynamic impairment that is different from extracranial dissection, which more often causes thromboembolism [1, 2]. IAD accounts for a small percentage (1–2%) of all ischemic strokes [3] and is classified according to modified TOAST criteria [3, 4]. It is, however, an important cause (10–20%) of stroke in young and middle-aged adults, especially in the Asian population [5, 6]. Diagnosis is challenging, as imaging features can be difficult to detect due to their small size and geometrical complexity. Multimodal imaging is, therefore, often required to confirm diagnoses.

Digital subtraction angiography (DSA) is the current gold standard for the characterization of IAD. It displays lumen structure and differentiates inflow from outflow if re-entrance is present. However, it cannot visualize wall structure, including hematoma. Multi-contrast high-resolution, cardiovascular magnetic resonance (hrCMR) has been increasingly used to characterize arterial abnormality by visualizing both lumen morphology and wall structure, including atherosclerosis, dissection and vasculitis [3, 7, 8]. Many pilot studies have demonstrated its complementary values for the diagnosis of intracranial dissection over DSA, magnetic resonance angiography (MRA) and computed tomography angiography (CTA) by harnessing its excellent soft tissue contrast [3, 9–11]. However, compared with carotid atherosclerosis, investigations on the diagnostic utility of hrCMR are much less commonly performed with intracranial vasculopathies including dissections [12]. It has been suggested that hrCMR should be used as a second-line diagnostic modality when there is diagnostic uncertainty [13].

Although encouraging results have been obtained supporting the use of hrCMR for the assessment of IAD, the morphological features and imaging characteristics of IAD remain unclear. Moreover, key determinates associated with patient clinical presentations remain largely unexplored. This study was designed to give a comprehensive understanding the geometric features of IAD and seeking those features associated with culprit and non-culprit lesions. Furthermore, lesions which had not been confirmed by DSA were analyzed to assess the value of hrCMR.

## Methods

### Study population

In this retrospective study, consecutive patients with IAD in Changhai Hospital, Shanghai, China between January 2014 and January 2019 underwent hrCMR, were screened by reviewing imaging examinations, laboratory tests, discharge report and other medical records, and data from those with DSA were used for analysis. This study was approved by the Institutional Review Board of Changhai Hospital and patients provided written informed consent. All patients had neurological symptoms within 30 days. Diffusion-weighted imaging (DWI) combined with hrCMR was performed within 7 days during hospitalization. Ischemic stroke was determined by the cerebral vascular neurological symptoms and positive DWI. The exclusion criteria were [14]: (1) dissection accompanied by other cerebral vasculopathies, including extracranial or intracranial atherosclerotic arteries with ≥ 50% stenosis, vasculitis, Moya-Moya disease, fibromuscular dysplasia; (2) chronic ischemic symptoms (> 12 weeks); (3) ascending aortic arch atheroma; (4) suspected cardio-embolic stroke; (5) previous strokes or transient ischemic attacks (TIA); (6) known coagulopathy or renal dysfunction (estimated glomerular filtration rate assessed by creatinine clearance < 60 mL/[min·1.73 m^2^]); and (7) clinical contraindications to CMR. The demographic and clinical characteristics, including age, sex, hypertension, hyperlipidemia, diabetes mellitus, smoking, and medication, were collected (Table 1).

**Table 1:** Comparison of patient demography between culprit and non-culprit dissections

	Mean ± SD or n (%)		
	Culprit n = 55	Non-culprit n = 20	*P* value
Male	45 (81.8)	17 (85.0)	0.748
Age	47.8 ± 12.4	59.2 ± 10.7	** < 0.001**
Age stage			
< 45 years	22 (40.0)	1 (5.0)	
45–65 years	28 (50.9)	12 (60.0)	
> 65 years	5 (9.1)	7 (35.0)	
Hypertension	48 (87.3)	5 (25.0)	** < 0.001**
Hyperlipidemia	15 (27.3)	5 (25.0)	0.844
Diabetes	9 (16.4)	7 (35.0)	0.081
Smoke	16 (29.1)	4 (20.0)	0.431
TG (mmol/L)	1.69 ± 1.73	1.71 ± 0.44	0.352
LDL (mmol/L)	2.44 ± 0.92	2.24 ± 0.86	0.410
HDL (mmol/L)	1.17 ± 0.35	1.16 ± 0.24	0.856

The bold values of *P* was considered statistical significance (< 0.05)

*HDL* high density lipoprotein, *LDL* low density lipoprotein, *TG* triglycerides

### High resolution cardiovascular magnetic resonance imaging acquisition

Patients were imaged using a 3 T whole body CMR system (Skyra; Siemens Healthineers, Erlangen, Germany) with a 20-channel phased array head and neck coil. In addition to whole brain CMR, after an initial multi-plane localizer sequence, an axial 3D time-of-flight (TOF) sequence was performed followed by 2D black blood fast-spin-echo (FSE) T2-weighted, 3D SPACE T1-weighted and gadolinium contrast enhanced 3D SPACE T1-weighted (CE-T1) sequences. Scan parameters were: T2 weighted sequence, slice thickness 2 mm, number of slices = 12, field of view (FOV) 100 × 100 mm^2^, matrix 256 × 320, in-plane resolution of 0.4 × 0.3 mm^2^, TR/TE = 2890 ms/46 ms, ETL = 20, NEX = 3; T1-weighted sequence, the spatial resolution was 0.5 mm isotropic, TR/TE = 1000 ms/17 ms, number of slices = 288, acquisition plane sagittal, FOV = 18 × 18 cm^2^, matrix 360 × 360, ETL = 60, echo spacing = 5.55 ms, echo train duration = 333 ms, bandwidth = 400 Hz/pixel, partial Fourier = 7/8, NEX = 2.

### Imaging analysis

All CMR images were initially analyzed independently by three readers (ZS was a resident with 5-year experience, XT was a neuroradiologist with more than 10-year experience and BT was a radiologist with 15-year experience on vessel wall imaging) blinded to the clinical information to assess the inter-observer reproducibility. XT redid the analysis two months later to assess the intra-observer reproducibility. The results were then disclosed to each other and any disagreement was resolved by consensus (the opinion from the senior reader BT would usually be more important).

Each detected dissection was independently classified as culprit or non-culprit with the consideration of brain DWI and location related to the vascular territory supplied by the branches distal to the dissection. Dissected vessels with a normal signal on DWI and the patients without classical cerebral vascular symptoms including, sensory or motor disorders, transient ischemic attack, or ischemic stroke, were considered to be non-culprit. Meanwhile, each dissection was classified into five different types (Type I: Classical dissection; Type II: Fusiform aneurysm; Type III: Long dissected aneurysm; Type IV: Huge aneurysm; Type V: Saccular aneurysm) in accordance with previous studies and consensuses [15, 16].

**Fig. 1:**
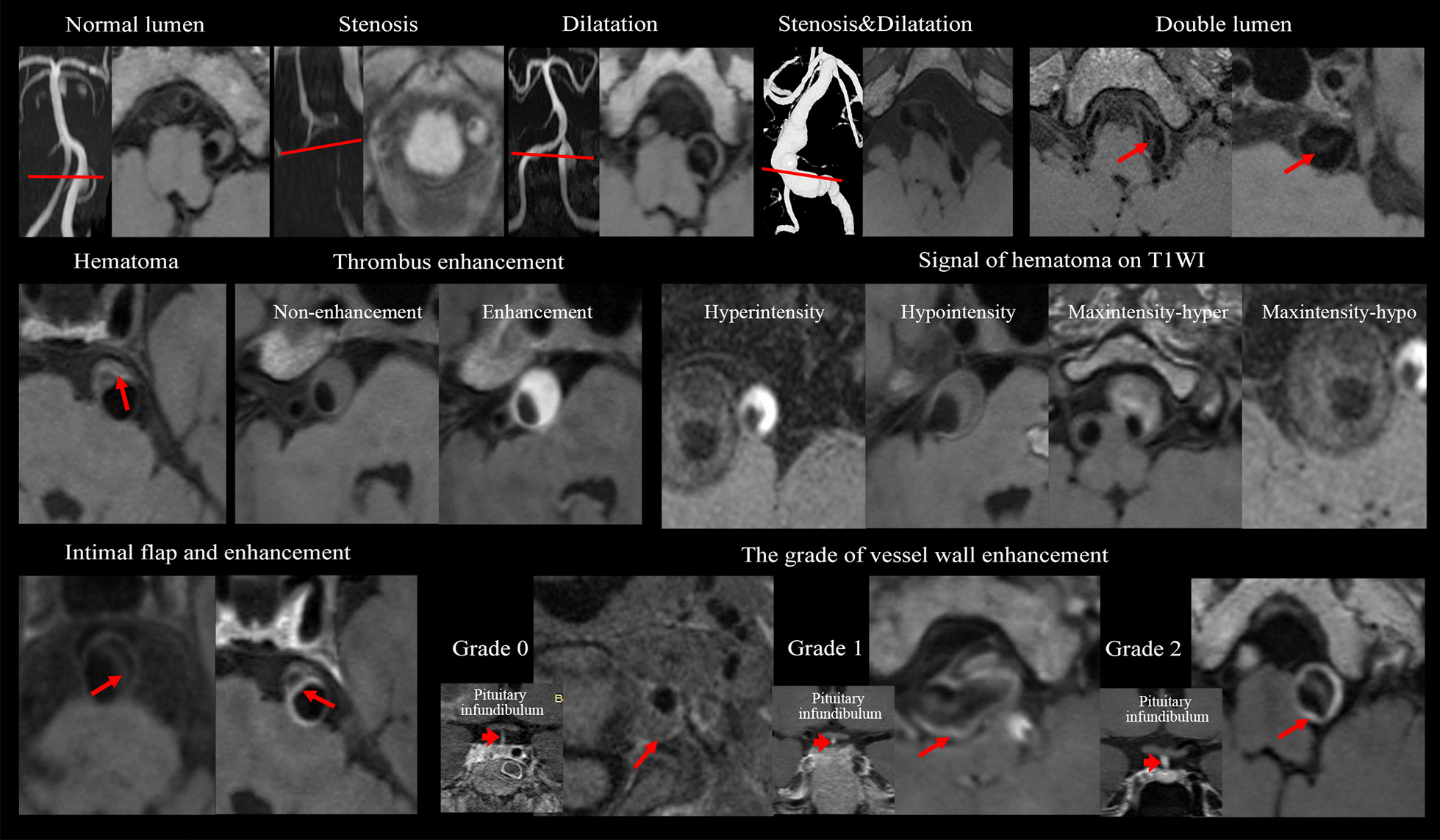
Representative high-resolution cardiovascular magnetic resonance (hrCMR) images of intracranial artery dissection (IAD) with different morphological and compositional features

All source images and curved multi-planar reformation were used to record: (1) lesion location (anterior or posterior circulation); (2) lumen shape; (3) presence or absence of hematoma; (4) the signal of hematoma; (5) presence or absence of a double lumen; (6) presence or absence of intimal flap; (7) presence or absence of intraluminal thrombus enhancement; (8) presence or absence of intimal flap enhancement; (9) the grade of vessel wall enhancement; and (10) the type of the dissection. Detailed definition of each of these characteristics can be found in the Additional file 1. Representative cases are shown in Fig. 1.

### Statistical analysis

All statistical analyses were performed using SPSS (version 24.0, Statistical Package for the Social Sciences, International Business Machines, Inc., Armonk, New York, USA). A normality test using the Shapiro–Wilk was performed to assess the distribution of each continuous variable. Univariate analysis was initially performed using t-tests, ANOVA or Mann–Whitney *U* tests where appropriate multivariate analysis was subsequently performed to include those with p < 0.10 in the univariate test. The odds ratios (OR) was calculated by a logistic regression model with stepwise selection of variables to determine independent predictors associated with lesion type. The diagnostic performance was described using receiver operating characteristic (ROC) curves and AUC values. The intra-observer and inter-observer agreement were evaluated by using the Cohen κ coefficient. Statistical significance was considered as p < 0.05.

## Results

### Patient demographics

A total of 101 patients met the inclusion criteria. Twenty-six patients were excluded due to chronic ischemic symptoms (n = 13), ≥ 50% stenosis of extracranial carotid atherosclerotic arteries (n = 8), vasculitis (n = 3), and previous stroke and TIA (n = 2). As a result, 75 patients (51.2 ± 12.9 years; 62 (83%) males) were included in the final analysis (55 culprit and 20 non-culprit). A detailed comparison of patient demography between individuals with and without culprit lesions is shown in Table 1. Compared with patients with non-culprit IAD, those with culprit lesions tended to be younger, vast majority (91%) under 65 years old, and had hypertension.

### Overall lesion characteristics on high resolution cardiovascular magnetic resonance imaging

**Fig. 2:**
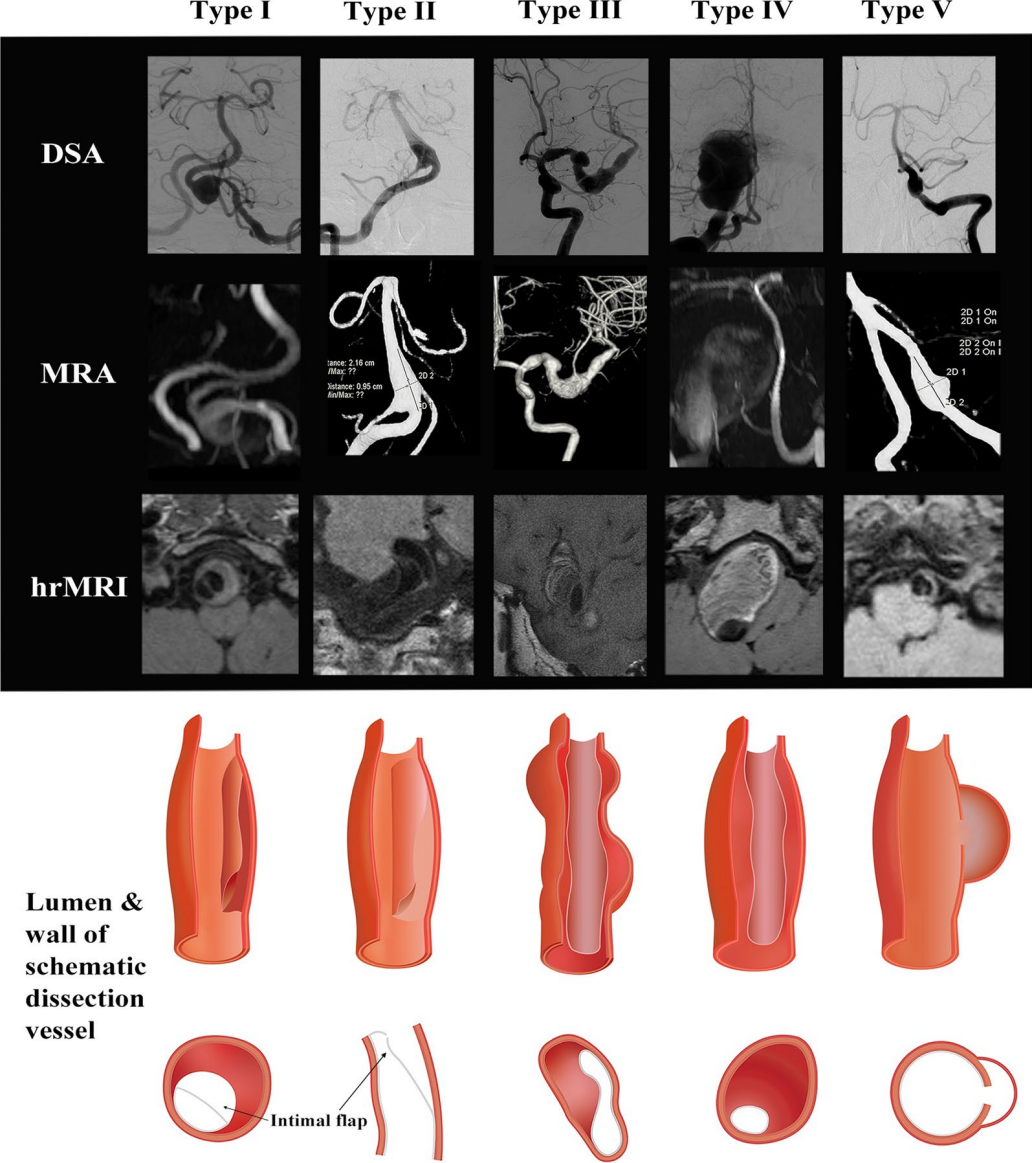
Different types of IAD with matched schematic drawings (longitudinal and cross-sectional view). Type I: Classical dissection; Type II: Fusiform aneurysm; Type III: Long dissected aneurysm; Type IV: Dolichoectatic dissecting aneurysm; Type V: Saccular aneurysm

**Fig. 3:**
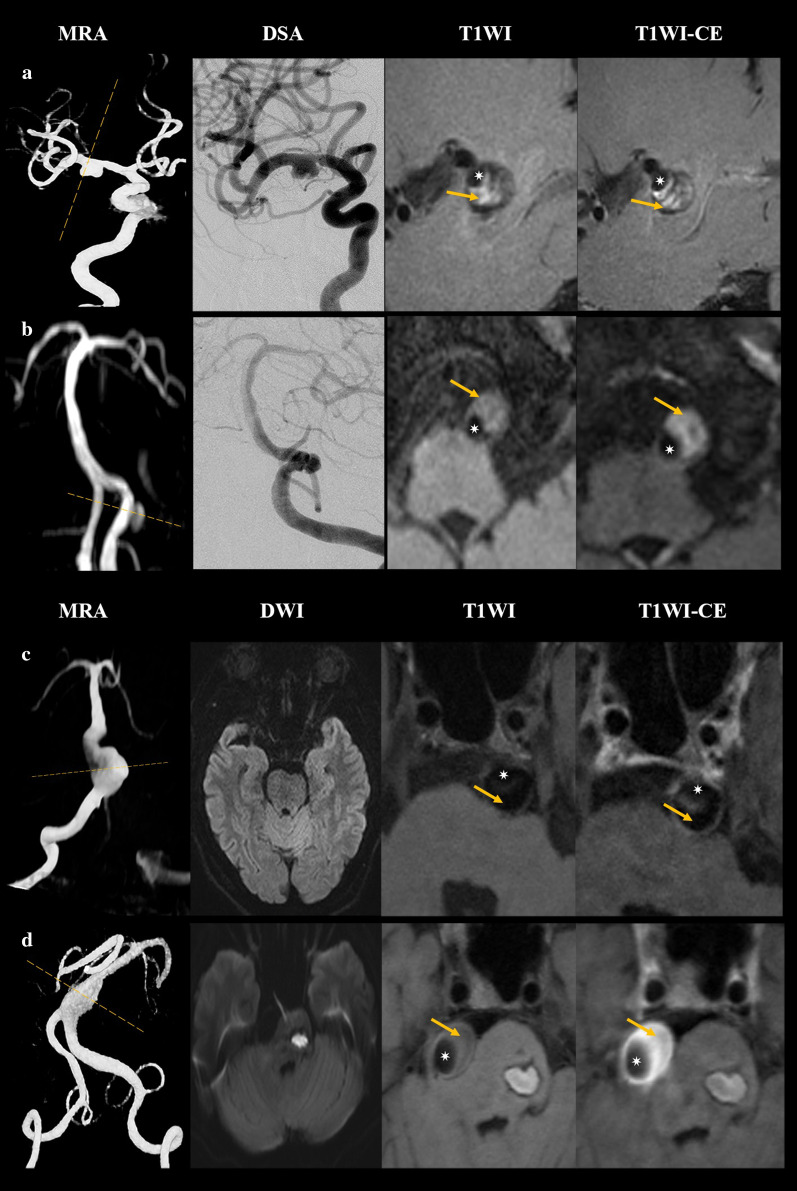
The cases of IAD. **a** a 51-year-old woman with acute transient ischemic attack (TIA). Both magnetic resonance angiography (MRA) and digital subtraction angiography (DSA) show an aneurysm with irregular surface on the M1 segment of right middle cerebral artery (MCA). T1-weighted and contrast enhanced (CE-T1) images show the lumen (yellow arrow) and pseudolumen (white star) on the right. **b** a 49-year-old man with acute ischemic symptom. Neither MRA nor DSA demonstrate an aneurysm or a dissection of the basilar artery (BA). T1-weighted and CE-T1 images show the lumen (yellow arrow) and pseudolumen (white star) on the right. **c** a 56-year-old man with dizziness and headache. MRA demonstrates an irregular aneurysm on the bottom of the BA, and no acute infarction was found in DWI. T1-weighted and T1-weighted enhancement (CE-T1) hrCMR are shown on the right. **d** a 44-year-old man with acute ischemic stroke on the left brainstem. MRA demonstrates an elliptic aneurysm on the BA, and DWI shows a sheet acute infarct in distribution of BA. T1-weighted and CE-T1 images are shown on the right

IADs involved in this study could be classified into five different types according to morphological features (Table 2 with schematic drawings and representative cases shown in Fig. 2): classical dissection [Type I; n = 50 (66.7%)], fusiform aneurysm [Type II; n = 1 (1.3%)], long dissected aneurysm [Type III; n = 3 (4.0%)], dolichoectatic dissecting aneurysm [Type IV; n = 9 (12.0%)] and saccular aneurysm [Type V; n = 12 (16.0%)]. Most lesions were classical dissections, followed by saccular and dolichoectatic dissecting aneurysm, while fusiform and long dissected aneurysms were uncommon in this study. Most lesions [n = 32 (88.9%)] were located in the vertebral artery with hematoma. Double lumen sign was visible in 48/75 (64.0%) lesions in hrCMR. However, such this was not visible in more than 40% of classical dissections, which might be the reason why 30% of classical dissections were not visualized in DSA (Fig. 3a, b). Intimal flap was visible in most of the lesions (93.3%). Enhancement on CE-T1 image was observed in most intimal flaps and vessel walls and the enhancement was also observed in the hematoma. Example images of patients with culprit and non-culprit dissection are shown in Fig. 3c, d.

**Table 2:** Clinical and radiological characteristics of the dissection among the five types

	Mean ± SD or n (%)					
	I: Classical dissection (n = 50)	II: Fusiform aneurysm (n = 1)	III: Long dissected aneurysm (n = 3)	IV: Dolichoectatic dissecting (n = 9)	V: Cysitical aneurysm (n = 12)	*P* value
Male	42 (84.0)	1 (100%)	3 (100%)	7 (77.8)	9 (75.0)	
Age	50.50 ± 11.31	71	40.67 ± 20.21	57.42 ± 11.90	57.42 ± 11.9	0.051
Hypertension	35 (70.0)	0 (0)	1 (33.3)	5 (55.6)	8 (66.7)	0.372
Hyperlipidemia	11 (22.0)	0 (0)	1 (33.3)	2 (22.2)	6 (50.0)	0.366
Diabetes	6 (12.0)	0 (0)	0 (0)	3 (33.3)	7 (58.3)	**0.010**
Smoking	10 (13.3)	0 (0)	1 (1.3)	4 (5.3)	5 (6.7)	0.347
TG (mmol/L)	1.62 ± 1.79	1.73	1.05 ± 0.38	1.49 ± 0.70	1.66 ± 0.81	0.978
LDL (mmol/L)	2.41 ± 1.02	1.99	2.41 ± 0.34	2.25 ± 0.72	2.39 ± 0.71	0.985
HDL (mmol/L)	1.21 ± 0.31	1.18	0.97 ± 0.05	0.92 ± 0.19	1.22 ± 0.41	0.170
Confirmed by DSA	35 (70.0)	1 (100)	2 (66.7)	8 (88.9)	12 (100)	**0.021**
Clinical symptoms	40 (80.0)	0 (0)	1 (33.3)	8 (88.9)	6 (50.0)	**0.037**
Location						**0.009**
ICA	7 (14.0)	0 (0)	0 (0)	0 (0)	0 (0)	
MCA	3 (6.0)	1 (100)	1 (33.3)	1 (11.1)	0 (0)	
VA	36 (72.0)	0 (0)	2 (66.7)	6 (66.7)	8 (66.7)	
BA	4 (8.0)	0 (0)	0 (0)	1 (11.1)	4 (33.3)	
PCA	0 (0)	0 (0)	0 (0)	1 (11.1)	0 (0)	
Lumen shape						** < 0.001**
Normal	11 (22.0)	0 (0)	0 (0)	0 (0)	2 (16.7)	
Stenosis	28 (56.0)	0 (0)	1 (33.3)	3 (33.3)	0 (0)	
Dilatation	11 (22.0)	1 (100)	1 (33.3)	6 (66.7)	10 (83.3)	
Stenosis and dilatation	0 (0)	0 (0)	1 (33.3)	0 (0)	0 (0)	
Hematoma	43 (86.0)	0 (0)	2 (66.7)	9 (100)	10 (83.3)	0.837
Hematoma signal on T1WI						**0.006**
Isointensity	7 (14.0)	1 (100)	1 (33.3)	0 (0)	2 (16.7)	
Hyperintensity	16 (32.0)	0 (0)	1 (33.3)	0 (0)	3 (25.0)	
Hypointensity	9 (18.0)	0 (0)	0 (0)	0 (0)	3 (25.0)	
Maxintensity	12 (24.0)	0 (0)	0 (0)	7 (77.8)	0 (0)	
Double lumen	29 (58.0)	1 (100)	3 (100)	6 (66.7)	9 (75.0)	0.284
Intimal flap	46 (92.0)	0 (0)	3 (100)	9 (100)	12 (100)	0.065
Thrombus enhancement	30 (60.0)	0 (0)	2 (66.7)	8 (88.9)	7 (58.3)	0.248
Intimal flap enhancement	39 (78.0)	0 (0)	3 (100)	8 (88.9)	9 (66.7)	0.259
Vessel wall enhancement grade						0.388
1: No enhancement	14 (28.0)	0 (0)	1 (33.3)	1 (11.1)	3 (25.0)	
2: Enhancement	14 (28.0)	1 (100)	2 (66.7)	2 (22.2)	3 (25.0)	
3: Obvious enhancement	22 (44.0)	0 (0)	0 (0)	6 (66.7)	6 (50.0)	

The bold values of *P* was considered statistical significance (< 0.05)

*BA* basilar artery, *DSA* digital subtraction angiography, *ICA* internal carotid artery, *MCA* middle cerebral artery, *PCA* posterior cerebral artery, *T1WI* T1 weighted imaging, *VA* vertebral artery

### Determinants associated with culprit and non-culprit lesions

**Fig. 4:**
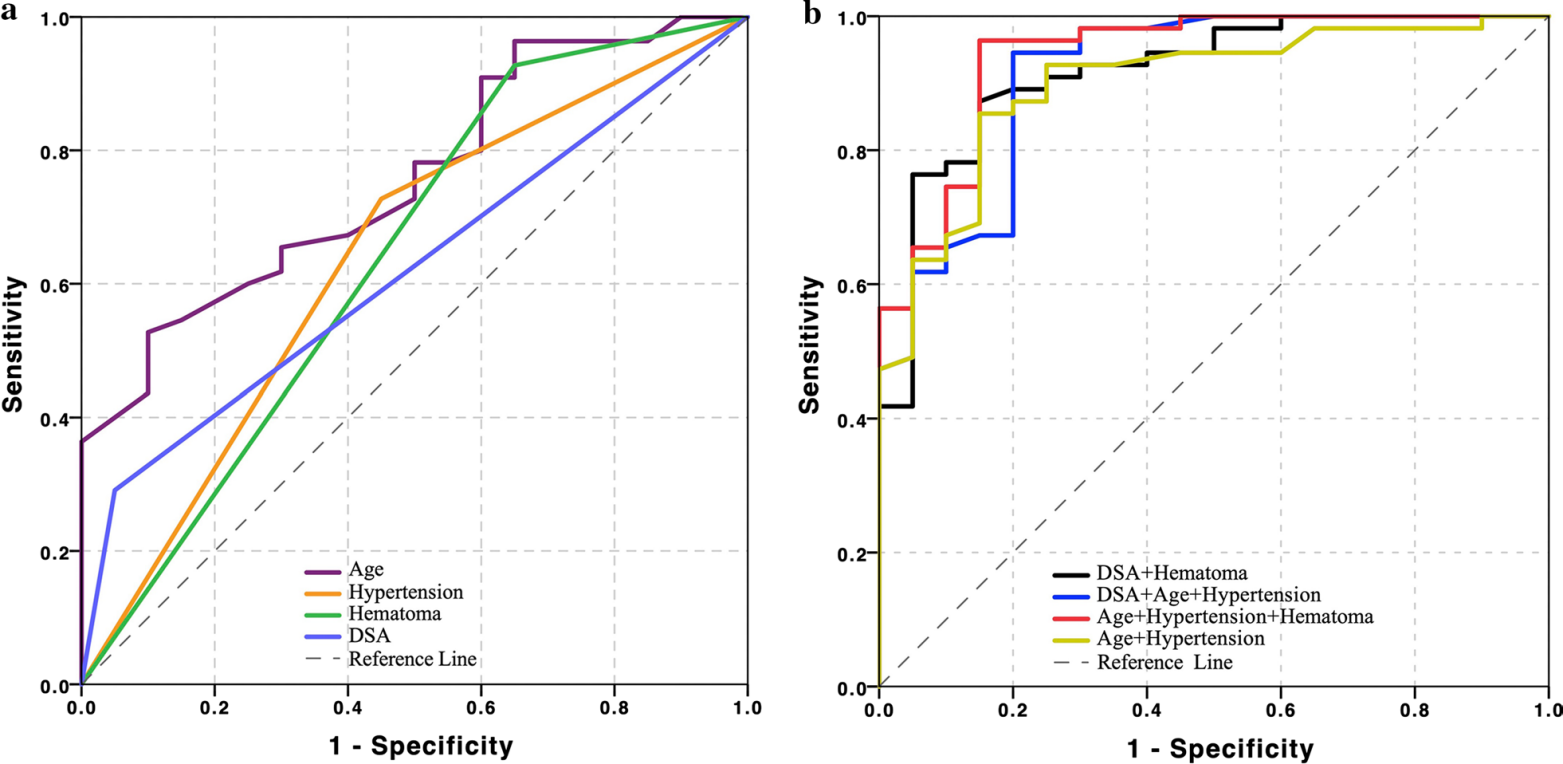
The power of different features and parameters in differentiating culprit and non-culprit lesions. **a** Four lower curves show the diagnostic performance of each independent parameters to identify the culprit IAD; **b** the four higher curves show diagnostic performance of combining the traditional features (age and hypertension) and DSA with or without hrCMR feature (hematoma) to differentiate the culprit dissection from non-culprit one, and the highest area under the curve (AUC) value of receiver operator characteristics (ROC) pooling traditional features and hrCMR feature was the AUC value of ROC pooling traditional features and hrCMR feature (0.940) is higher than that of ROC from DSA + hrCMR (0.918)

Due to the limited case number, Type II (n = 1) and III (n = 3) lesions were excluded for the statistical analysis. As listed in Tables 1 and 3, univariate analysis showed that age (p < 0.001), hypertension (p < 0.001), diabetes (p = 0.081), location (p = 0.073), hematoma (p = 0.003), hematoma signal on T1WI (p < 0.001), and dissection type (p = 0.037) were associated with ischemic events caused by the culprit dissection. Further multivariate logistic regression showed that age (OR, 0.831; 95% confidence interval (CI), 0.752–0.919; p < 0.001), hypertension (OR, 66.620; 95% CI 5.909–751.108; p = 0.001) and hematoma in hrCMR (OR, 16.803; 95% CI 1.006–280.806; p = 0.037) were significantly associated with the culprit dissection with area under the curve (AUC) being 0.757, 0.639 and 0.641, respectively (Fig. 4a). The AUC value was improved to 0.898 when traditional risk features (age and hypertension) were combined (Fig. 4b). If DSA was only considered, the AUC was 0.620. The AUC was improved to 0.918 when DSA and hrCMR were combined (Fig. 4b).

**Table 3:** Comparison of lesion features between culprit and non-culprit dissections

	n (%)		
	Culprit n = 55	Non-culprit n = 20	P value
Lesion location			0.073
ICA	6 (10.9)	1 (5.0)	
MCA	5 (9.1)	1 (5.0)	
VA	40 (72.7)	12 (60.0)	
BA	3 (5.5)	6 (30.0)	
PCA	1 (1.8)	0 (0)	
Lumen shape			0.124
Normal	9 (16.4)	4 (20.0)	
Stenosis	27 (49.1)	5 (25.0)	
Dilatation	19 (34.5)	10 (50.0)	
Stenosis and dilatation	0 (0)	1 (5.0)	
Hematoma	51 (92.7)	13 (65.0)	**0.003**
Hematoma signal on T1WI			**0.003**
Isointensity	4 (7.3)	7 (35.0)	
Hyperintensity	19 (34.5)	1 (5.0)	
Hypointensity	7 (12.7)	5 (25.0)	
Heterogeneous intensity	25 (45.5)	7 (35.0)	
Double lumen	34 (61.8)	14 (70.0)	0.514
Intimal flap	51 (92.7)	19 (95.0)	0.727
Thrombus enhancement	37 (67.3)	10 (20.0)	0.171
Intimal flap enhancement	42 (76.4)	17 (85.0)	0.419
Vessel wall enhancement grade			0.739
1: No enhancement	15 (27.3)	4 (20.0)	
2: Enhancement	15 (27.3)	7 (35.0)	
3: Obvious enhancement	25 (45.5)	9 (45.0)	

The bold values of *P* was considered statistical significance (< 0.05)

### Characteristics of intracranial artery dissection lesions visible only on hrCMR

IAD was diagnosed in 17 out of 75 cases (16 of which were judged to be culprit) were not visible on DSA and diagnosed only with hrCMR. 4 lesions showed normal luminal dimension, 10 narrowing, 2 dilation and one mixture of narrowing and dilation (Additional file 1: Table S1). Most of these cases (n = 15) were Type I (classical dissection), one was Type III (long dissected aneurysm) and one was Type IV (dolichoectatic dissecting aneurysm). Hematoma was visible in 14 cases, intimal flap was found in 15 cases and double lumen sign was found in 10 cases in hrCMR. Six lesions were located in the anterior circulation [internal carotid artery (ICA) and middle cerebral artery (MCA)], and 11 in the posterior circulation [vertebral artery (VA), basilar artery (BA) and posterior cerebral arteries (PCA)].

### Reproducibility of hrCMR readings

The intra- and inter-observer reproducibility was excellent. The Kappa value was 0.912 [0.847, 0.983] (mean [range]) for the intra-observer agreement in identifying lesion features using hrMRI and the Kappa value for the inter-observer reproducibility was 0.903 [0.826, 0.975]. More information about each feature can be found in Additional file 1: Table S2.

## Discussion

The analysis found that IAD could be classified into five different types according to morphological features in hrCMR. Age, hypertension and hematoma visualized in hrCMR could effectively differentiate culprit and non-culprit lesions. Moreover, compared with pooled traditional risk factors, the ROC curve is significantly higher when combining traditional risk features and hrCMR characteristics (hematoma). This study demonstrated a significant incremental value of hrCMR in identifying culprit IAD over traditional risk factors, in particular for those which could not be diagnosed using DSA.

Previous studies have reported [1, 2] that more than 70% of patients with cervicocerebral artery dissection presented as ischemic stroke, and it accounts for approximately 15% of ischemic strokes in young patients (15–49 years). This high percentage was confirmed by our study, in that young patients tended to have more recent neurological symptoms (29.3% of patients with culprit dissection were less than 45 years in this study). Recent research has found hypertension to be an important predictive factor for VA dissection [14], while Shin et al. [17] reported that compared with patient with cervical artery dissection, there was a higher prevalence of hypertension in those with IAD. This study agreed with previous findings that hypertension was significantly associated with culprit IAD.

Hematoma on the intraluminal vessel wall is a common pathogenomic feature which can be found in more than 50% of IADs [5]. This study confirmed the high prevalence of hematoma in IAD, and it was noticeably more common in the culprit lesions than non-culprit. However, the association between the presence of hematoma and patient clinical presentation remains unclear. It has been reported that hematoma in cervical artery dissection was not associated with ischemic stroke [14] and an investigation with a smaller patient number (n = 25) found hematoma in IAD to not have any relationship with neurological symptoms [18]. It was also reported that hematoma was commonly found in symptomatic patients with intracranial vertebrobasilar dissections as detected by T1-weighted imaging [19]. Moreover, the hematoma has been found in lesions in patients with acute symptoms as evidenced by tissue samples obtained from surgery or autopsy [20].

Currently, DSA remains the better method for identifying and characterizing artery dissections, especially in extracranial and intracranial arteries [2, 5]. However, it only delineates the lumen condition, such as in the visualization of pseudoaneurysm, stenosis and double lumen. This lumen-only delineation may limit its capacity for the detection of lesions with hematoma but without a re-entry, such as the Type I lesion considered in this study. In total, 17 IADs were diagnosed according to hrCMR, but were not confirmed in DSA, among which 15 were Type I (which was 30% of all Type I lesions). This suggests an important complementary value of hrCMR over the luminal condition assessment in the identification of IAD.

Compared with conventional DSA, MRA or CTA, hrCMR can provide far greater lesion detail. For instance, by referring to the signal intensity on T1-weighted and T2-weighted images, hematoma can be classified as hyperacute (< 24 h), acute (1–3 days), early subacute (> 3 days), late subacute (> 7 days) and chronic (> 14 days). If the hematoma appears homogeneously on CMR, the dissection might have formed at a single instance, whereas a heterogeneous appearance might imply a recurrent development. With the assist of contrast agents, local inflammation and the existence of vasa vasorum can be assessed via vessel wall enhancement [21, 22]. The enhancement also differentiates the lesion stage [8, 23] for example, the enhancement tended to be lower from the earlier stages to the chronic stage. Persistent vessel wall enhancement may imply intimal hyperplasia, granulation tissue or the vasa vasorum development during the healing process [24]. However, no significant difference was found in this study when the enhancement was compared between culprit and non-culprit lesions. Considering the advantage of demonstrating luminal stenosis, intramural hematoma and outer wall boundaries, its high accuracy in IAD detection and its non-invasive nature, hrCMR should be used as a routine imaging protocol prior to invasive DSA.

### Limitations

Despite interesting findings, the following limitations exist: (1) this is a single-center study; (2) the sample size is relatively small, in particular, the number cases of Type II and III lesions; (3) due to the small lesion dimension and the resolution limits of hrCMR, detailed lesion compositional features were not characterized and analyzed.

## Conclusion

hrCMRI is useful for visualizing and characterizing IAD, and provide a significant complementary value over DSA for the diagnosis of IAD.

## Supplementary Information

**Additional file 1: Table S1.** Clinical and radiological characteristics of the dissection. **Table S2.** Reproducibility of HRMRI measurement.

## Data Availability

All data generated or analyzed during this study are included in this published article.

## Abbreviations

AUC: Area under curve
BA: Basilar artery
CE-T1: Contrast enhanced T1-weighted
CI: Confidence interval
CTA: Computed tomography angiography
DSA: Digital subtraction angiography
DWI: Diffusion-weighted imaging
FOV: Field of view
FSE: Fast spin echo
hrCMR: High-resolution cardiovascular magnetic resonance
IAD: Intracranial artery dissection
ICA: Internal carotid artery
ICC: Intra-class coefficient
IPH: Intraplaque hemorrhage
MCA: Middle cerebral artery
MRA: Magnetic resonance angiology
OR: Odds ratio
PCA: Posterior cerebral arteries
ROC: Receiver operating characteristic
TIA: Transient ischemic attack
TOF: Time of flight
VA: Vertebral artery
