# Supplementary material for: Identification of high risk clinical and imaging features for intracranial artery dissection using high-resolution cardiovascular magnetic resonance
Source: J Cardiovasc Magn Reson. 2021 Jun 14;23:74. doi: 10.1186/s12968-021-00766-9 (PMC8201847; doi:10.1186/s12968-021-00766-9)
Supplement: Supplementary file 1 — Additional file 1: Table S1. Clinical and radiological characteristics of the dissection. Table S2. Reproducibility of HRMRI measurement. [file 12968_2021_766_MOESM1_ESM.docx]

**Supplemental Materials**

**Title:** Identification of high risk clinical and imaging features for intracranial artery dissection using high-resolution MRI

**Authors:**

Zhang Shi, MD^1,2*^; Xia Tian, MD^1*^; Bing Tian, MD^1#^; Zakaria Meddings, MEng^2^; Xuefeng Zhang, MD^1^; Jing Li, MD^1^; David Saloner, PhD^3^; Qi Liu, MD^1^; Zhongzhao Teng, PhD^2,4^; Jianping Lu, MD^1^^#^

**Affiliations:**

1. Department of Radiology, Changhai Hospital, Naval Medical University, Shanghai, China

2. Department of Radiology, Addenbrooks’ Hospital, University of Cambridge, Cambridge, UK

3. Department of Radiology and Biomedical Imaging, UCSF, San Francisco, CA, USA

4. Beijing Advanced Innovation Center for Biomedical Engineering, Beihang University, Beijing, China

^*^ Equal contribution

^#^ Corresponding authors

**Definition of the characteristics on hrMRI**

(1) The location of intracranial dissected vessels:

*Anterior circulation: middle cerebral artery (MCA), internal carotid artery (ICA)*

*Posterior circulation: basilar artery (BA), vertebral artery (VA)*

(2) The shape of lumen:

*Normal: the diameter of the lumen on the lesion is as similar as the reference lumen*

*Stenosis: the diameter of the lumen on the lesion is smaller than the reference lumen*

*Dilatation: the diameter of the lumen on the lesion is larger than the reference lumen*

*Stenosis&dilatation: there are both stenosis lumen and dilatated lumen on the lesion*

(3) Hematoma:

*crescent-shaped thickening of the arterial wall that was isointense or hyperintense on precontrast images.*

(4) Hematoma signal on T1WI:

*Isointensity: the intensity is similar to that of normal muscle nearby the artery*

*Hyperintensity: the intensity is greater than that of normal muscle nearby the artery*

*Hypointensity: the intensity is similar or less than that of normal muscle nearby the artery*

*Maxintensity: there are both hyperintensity and hypointensity*

(5) Double lumen:

*blood flow that was divided into a true and a false lumen.*

(6) Intimal flap:

*a curvilinear and isointense line crossing the flow void lumen or between a hyperintense hematoma that extended to the sidewall.*

(7) Thrombus enhancement:

*the thrombus on the intraluminal well with isointensity or maxintensity on pre-contrast images, as well as an area of intraluminal contrast enhancement on postcontrast images.*

(8) Intimal flap enhancement:

*intimal flap with hyperintensity on postcontrast images compared with the pre-contrast images.*

(9) Grade of vessel wall enhancement

*Grade 0: no enhancement, similar to that of normal vessel walls*

*Grade 1: enhancement, greater than that of normal intracranial arteries wall but less than that of pituitary* *infundibulum*

*Grade 2: obvious enhancement, similar to or greater than that of pituitary infundibulum*

(10) Type of dissection

*Type-I (Classical dissection)*

*Type-II (Fusiform aneurysm)*

*Type-III (Long dissected aneurysm)*

*Type-IV (Dolichoectatic dissecting aneurysm)*

*Type-V (Saccular aneurysm)*

**Table S1.** Clinical and Radiological Characteristics of the dissection

|  | n (%) | | |
| --- | --- | --- | --- |
|  | Agree with DSA  n=58 | Disagree with DSA n=17 | *P* value |
| Clinical symptoms | 39 (67.2) | 16 (94.1) | **0.028** |
| Radiological characteristics |  |  |  |
| Location |  |  | **0.026** |
| Anterior circulation (ICA&MCA) | 7 (12.1) | 6 (35.3) |  |
| Posterior circulation (VA, BA, and PCA) | 51 (87.9) | 11 (64.7) |  |
| Lumen shape |  |  | **0.019** |
| Normal | 9 (15.5) | 4 (23.5) |  |
| Stenosis | 22 (37.9) | 10 (58.8) |  |
| Dilatation | 27 (46.6) | 2 (11.8) |  |
| Stenosis&Dilatation | 0 (0) | 1 (5.9) |  |
| Hematoma | 50 (86.2) | 14 (82.4) | 0.693 |
| Hematoma signal on T1WI |  |  | 0.639 |
| Isointensity | 8 (13.8) | 3 (17.6) |  |
| Hyperintensity | 14 (24.1) | 6 (35.3) |  |
| Hypointensity | 9 (15.5) | 3 (17.6) |  |
| Maxintensity | 27 (46.6) | 5 (29.4) |  |
| double lumen | 38 (65.5) | 10 (58.8) | 0.613 |
| intimal flap | 55 (94.8) | 15 (88.2) | 0.338 |
| Thrombus enhancement | 36 (62.1) | 11 (64.7) | 0.843 |
| intimal flap enhancement | 47 (81.0) | 12 (70.6) | 0.355 |
| Vessel wall enhancement grade |  |  | **0.005** |
| No enhancement | 10 (17.2) | 9 (52.9) |  |
| Enhancement | 18 (31.0) | 4 (23.5) |  |
| Obvious enhancement | 30 (51.7) | 4 (23.5) |  |
| Type of the dissection |  |  | **0.021** |
| I: Classical dissection | 35 (60.3) | 15 (88.2) |  |
| II: Fusiform aneurysm | 1 (1.7) | 0 (0) |  |
| III: Long dissected aneurysm | 2 (3.4) | 1 (5.9) |  |
| IV: Dolichoectatic dissecting | 8 (13.8) | 1 (5.9) |  |
| V: Saccular aneurysm | 12 (20.7) | 0 (0) |  |

*The bold values of p was considered statistical significance (<0.05).*

**Table S2**  Reproducibility of CMR measurement

|  | Intra-observer | Inter-observer |
| --- | --- | --- |
| Location | 0.983 | 0.975 |
| Lumen | 0.932 | 0.847 |
| intraluminal hematoma | 0.937 | 0.903 |
| intraluminal hematoma Signal | 0.928 | 0.914 |
| double lumen | 0.927 | 0.886 |
| intimal flap | 0.915 | 0.887 |
| Intraluminal thrombus enhancement | 0.904 | 0.877 |
| intimal flap enhancement | 0.889 | 0.862 |
| Vessel wall enhancement grade | 0.928 | 0.946 |
| Type of the dissection | 0.898 | 0.882 |
